# Supplementary material for: Effects of lower and upper body fatigue in striking response time of amateur karate athletes
Source: PeerJ. 2023 Jan 31;11:e14764. doi: 10.7717/peerj.14764 (PMC9897062; doi:10.7717/peerj.14764)

Figure S1.

Legend:

LB-Punch: Lower body fatigue followed by punch and kick RT, respectively.

LB-Kick: Lower body fatigue followed by kick and punch RT, respectively.

UB-Punch: Upper body fatigue followed by punch and kick RT, respectively.

UB-Kick: Upper body fatigue followed by kick and punch RT, respectively.

**A**. Mean punch RT and confidence intervals for each experimental condition, stratified by experience level.

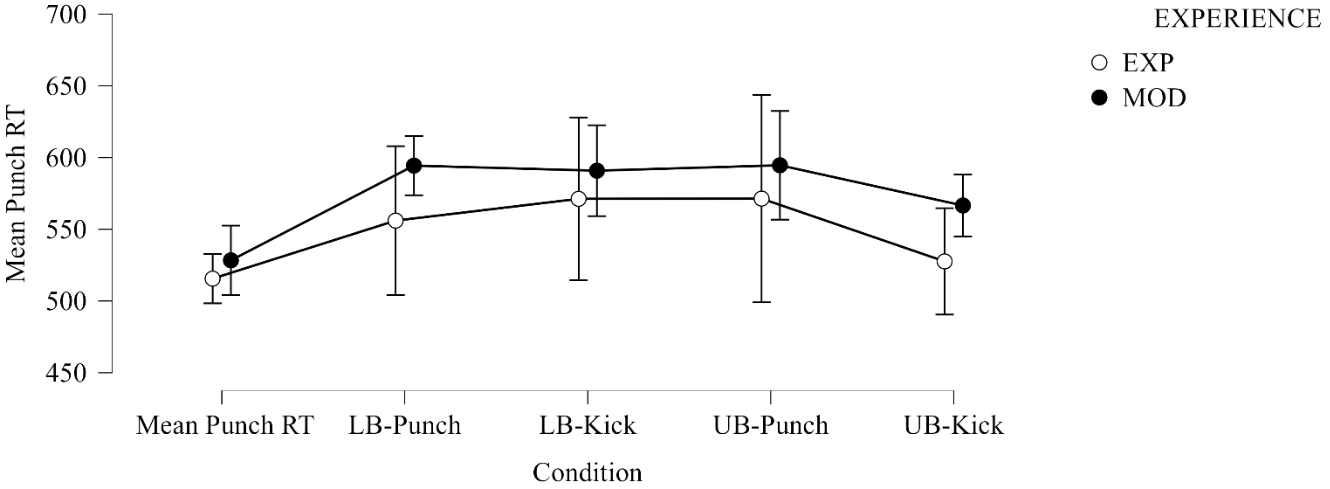


**B**. Mean punch RT and confidence intervals for each experimental condition, stratified by sex.

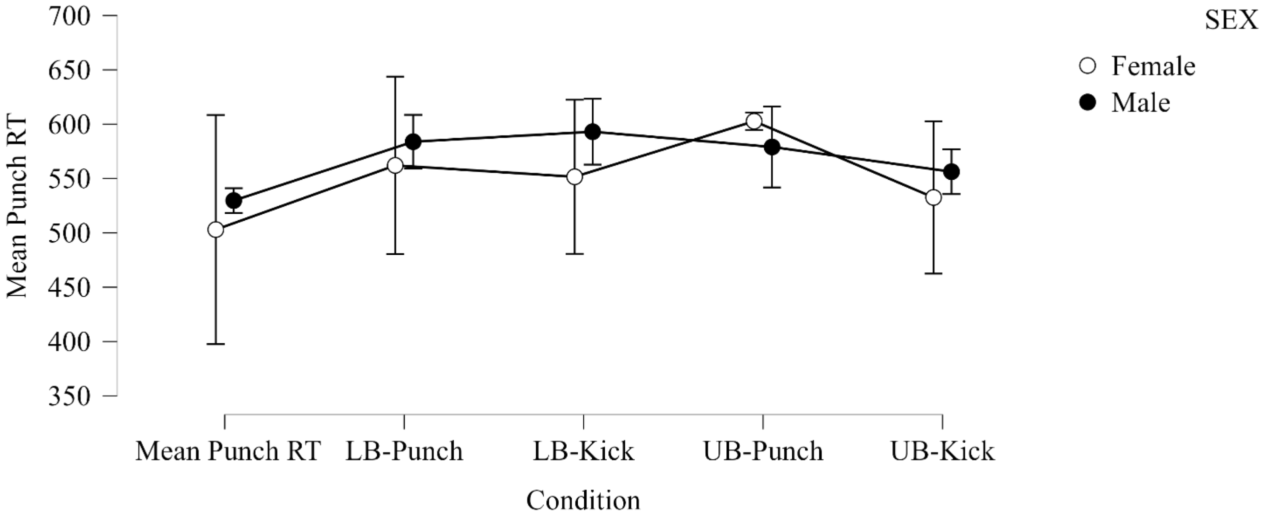

**C**. Mean kick RT and confidence intervals for each experimental condition, stratified by experience level.

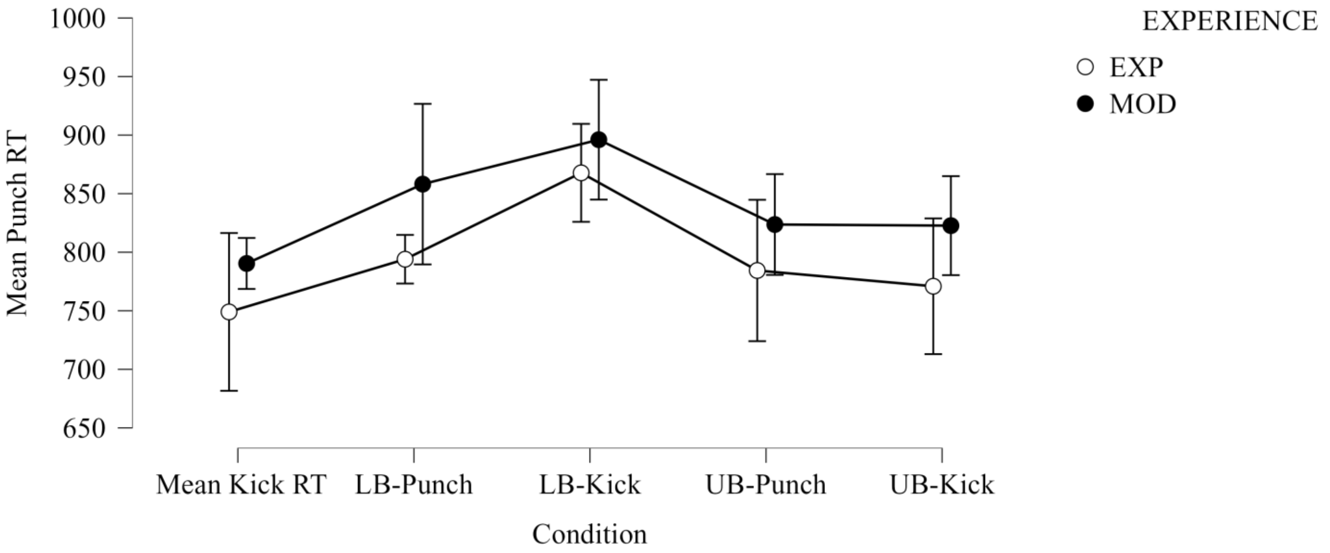


**D**. Mean punch RT and confidence intervals for each experimental condition, stratified by sex.

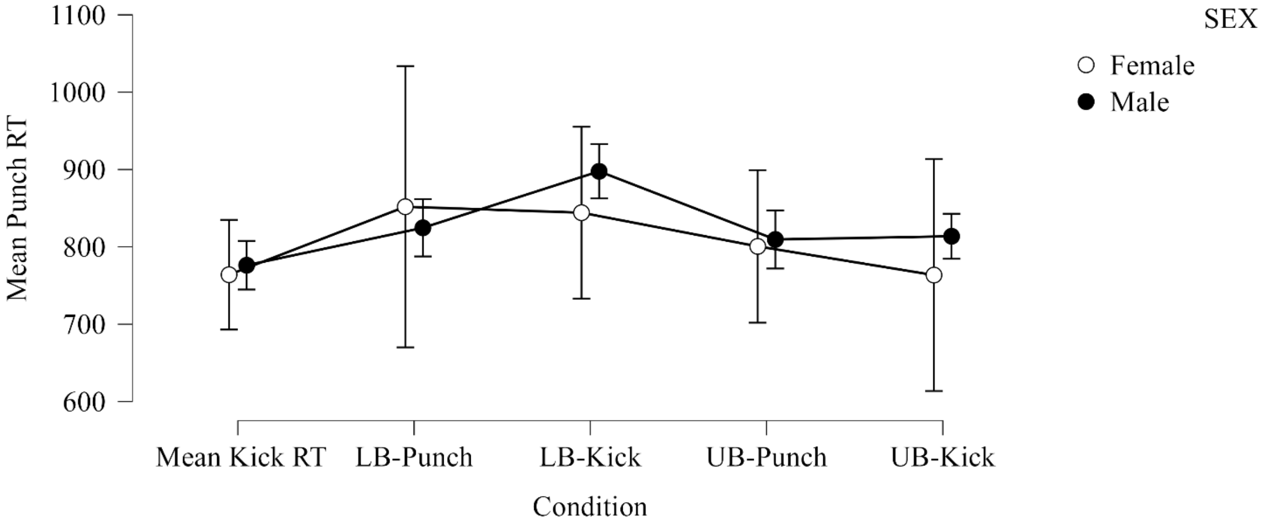

Supplement: Supplemental Information 2 — LB-Punch, Lower body fatigue followed by punch and kick RT, respectively. LB-Kick, Lower body fatigue followed by kick and punch RT, respectively. UB-Punch, Upper body fatigue followed by punch and kick RT, respectively. UB-Kick, Upper body fatigue followed by kick and punch RT, respectively. [file peerj-11-14764-s002.pdf]
